# Interactions in Bacterial Biofilm Development: A Structural Perspective

**DOI:** 10.2174/138920312804871166

**Published:** 2012-12

**Authors:** James A Garnett, Steve Matthews

**Affiliations:** Centre for Structural Biology, Department of Life Sciences, Imperial College London, South Kensington, London SW7 2AZ, UK

**Keywords:** Biofilm, adhesion, dispersin, structural biology.

## Abstract

A community-based life style is the normal mode of growth and survival for many bacterial species. These cellular accretions or biofilms are initiated upon recognition of solid phases by cell surface exposed adhesive moieties. Further cell-cell interactions, cell signalling and bacterial replication leads to the establishment of dense populations encapsulated in a mainly self-produced extracellular matrix; this comprises a complex mixture of macromolecules. These fascinating architectures protect the inhabitants from radiation damage, dehydration, pH fluctuations and antimicrobial compounds. As such they can cause bacterial persistence in disease and problems in industrial applications. In this review we discuss the current understandings of these initial biofilm-forming processes based on structural data. We also briefly describe latter biofilm maturation and dispersal events, which although lack high-resolution insights, are the present focus for many structural biologists working in this field. Finally we give an overview of modern techniques aimed at preventing and disrupting problem biofilms.

## 1 INTRODUCTION

The ability of a biological cell to sense and respond to its local environment underlies the existence of every living organism. Furthermore, communication between sibling and rival cells has led to the evolution of multicellular, higher ordered life. For many years it was believed that bacteria inhabit the planet exclusively in a planktonic form, as free-living cells, but it is now widely accepted that most microorganisms reside primarily in biofilms [1-4]. A biofilm is a complex agglomeration of genetically similar or distinct cells adhered to a solid surface and to one another, encased in a scaffold of self-produced extracellular polymeric substances (EPS). In their mature form they often appear as pyramid or mushroom-like structures, which are embedded with cavities and channels allowing the sequestration of water together with the exchange of nutrients and waste [5]. 

Bacterial biofilms are ubiquitous in the environment and can be found on almost any hydrated non-shedding surface; including rivers, stagnant pools, man-made materials and biological matter. Whilst many of these communities can be used for the synthesis of valuable products and the treatment of waste, others can cause problems in industrial applications and/or persistent virulence in humans and other animals. These bacterial aggregates provide protection from a wide range of environmental factors such as fluctuating pH [6], exposure to UV light [7], dehydration [8] and antimicrobial agents [9,10]. Biofilms have been identified as far back as 3.2-billion years ago [11] and it has been suggested that rather than the free living cells developing into a more complex sessile form, planktonic and biofilm phenotypes have evolved together [12].

**Fig. (1):**
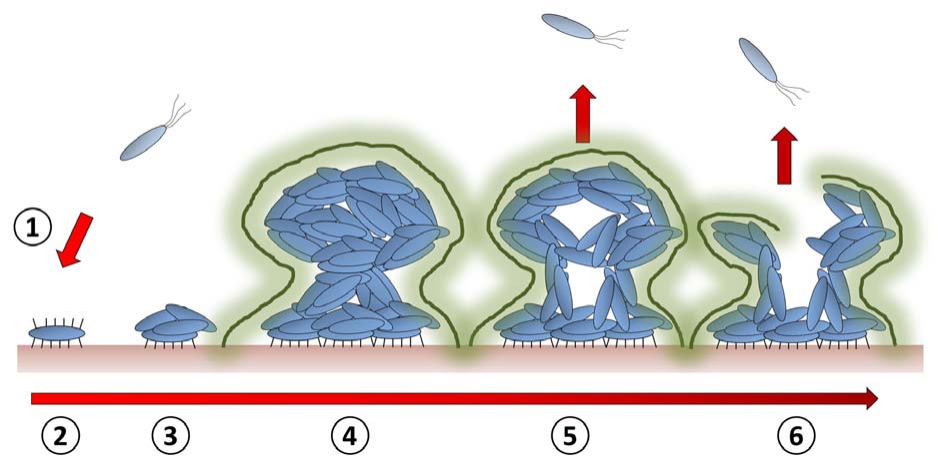
**Schematic representation of the biofilm life cycle.** (1) Free swimming bacteria (2) adhere to a surface using cell surface displayed adhesin molecules. (3) Bacteria begin to divide and the expression of further macromolecules allows them to stick together in small microcolonies. (4) As these colonies grow they begin to secrete a complex mixture of carbohydrates, protein and lipids that encapsulates the bacteria. This biofilm matrix (fuzzy outline) provides protection and stability for the maturing biofilm. (5) When the biofilm reaches maturity, a number of factors will have developed a heterogeneous arrangement of cells and molecules within the biofilm, and given rise to solvent filled cavities and channels. This can lead to dispersal of cells from the cellular mass. (6) Upon signal from the environment (waste build up or demand for nutrients, for example), molecules are released that cause cell lysis and matrix dissemination. Many planktonic cells are now released and can find a new habitat.

The biofilm life-cycle involves several stages (Fig. **1**) [5]. If we start with a single mobile bacterium, we can consider the first stage of this process to be cellular adhesion to a solid surface [13]. This initial event is highly reversible, although upon cues from the environment the production of additional adhesion molecules can be initiated and attachment becomes more tenacious [13,14]. These cells begin to divide and signals from neighbouring bacteria lead to changes in phenotype and the display of further proteinaceous molecules on their surface, instigating protein/polysaccharide-mediated inter-bacterial interactions. Further sensing of the local environment leads to the secretion of a sticky matrix of extracellular polymeric substances (EPS); comprising polysaccharides, proteins, nucleic acids and lipids [14]. These components allow the three dimensional architecture of the mature biofilm to form, whilst also increasing adhesion between bacteria and with the surface support. At this stage inter-cellular communication leads to synergy within the community [12,15], with the diffusion of oxygen/nutrients inwards and waste/signals outwards. The heterogeneous distribution of cells within the biofilm is in part orchestrated by dispersins [16]. These reagents act as surfactants or enzymes that disrupt interbacterial interactions, producing water filled channels and lead to the active and passive dispersal of cells from the biofilm mass. This cycle is generally accepted as a developmental process, where a hierarchy of genetic events controls the transition in response to environmental cues such as quorum sensing; although this is still very much a working model and further validation is required [17].

Biofilms are a very attractive area of research as they effect many industrial processes, are a major cause of antibiotic resistance and harbour the potential for novel technologies and materials. In this review we will be concentrating on the architectural protein components of these bacterial accretions and how they mediate persistent host-pathogen interactions. We will then give a brief discussion of therapeutic targets with the aim of dismantling and/or removing problematic biofilms. 

## 2 INITIAL SURFACE ADHESION

The initial attachment of prokaryotes to abiotic and biological surfaces is by far the most understood process of biofilm development. The bacterial surface is decorated with an arsenal of diverse and often multifunctional macromolecules. Adhesive structures are projected away from the cell surface with sufficient distance to sample the surrounding environment and recognize appropriate interactions. At the time of this review, a search of the protein data bank (PDB: www.pdb.org) revealed 140 ‘adhesin’ structures from bacteria, many of which are unique proteins. In this section a number of different fibres from a range of bacteria will be discussed, although the focus will be on those structures that contribute to a well-defined understanding of surface attachment. For example, whilst type IV pili (T4P) are very important factors leading to surface attachment in many bacteria, no receptor complexes are available and so they will not be discussed here. In addition, although the chaperone-usher (CU) assembled type I and P pili are important lectins from *Escherichia coli*, a wealth of both structural and biochemical information has already been amassed and a number of excellent articles and reviews are already available [18-22], so again these will not be described. 

### 2.1 MSCRAMMs 

*Staphylococcus aureus *is a regular commensal of the skin and anterior nares of animals and humans [23]. Whilst harmless in these environments, *S. aureus *may become a dangerous pathogen if it crosses the epithelial barrier where it can infect almost any organ and cause abscesses, pneumonia, endocarditis, sepsis and infections associated with medical implants [24]. A number of surface components of *S. aureus* have been implicated in biofilm formation. The exopolysaccharide biofilm matrix is composed of a polymer of poly-N-acetyl-β-(1-6)-glucosamine, termed polysaccharide intercellular adhesin (PIA) or poly-N-acetylglucosamine (PNAG) and mediates intercellular adhesion events [25,26]. *S. aureus* biofilms can also form independently of PIA and the composition of this proteinaceous matrix includes SasG [27], Protein A (Spa) [28,29], fibronectin binding proteins (FnBPs) [30,31] and biofilm associated protein (Bap) [32]. SasG can bind desquamated nasal epithelial cells [31] and promotes inter-bacterial aggregation [33]. Spa binds immunoglobulin’s which enables *S. aureus* to evade innate/adaptive immune responses [34] and can promote multicellular adherence [28]. FnBPs have been shown to mediate intercellular biofilm interactions [30] [31], recognize fibronectin and fibrinogen [35,36] and can also promote the invasion of epithelial cells [37]. Bap is involved in the initial attachment to inert surfaces/interbacterial interactions [32] and also prevents cellular internalization of *S. aureus* through binding the GP96 host receptor, which interferes with the FnBP mediated invasion pathway [38].

Spa and FnBPs belong to a large family of proteins called MSCRAMMs (microbial surface components recognizing adhesive matrix molecules) [39] that enable many pathogenic Gram-positive bacteria to interact with the eukaryotic extracellular matrix (ECM). The ECM is a biologically active material that encapsulates eukaryotic cells and contains a mixture of macromolecules including collagen, fibronectin and fibrinogen, and functions in both cellular structure and physiology. A number of MSCRAMMs are involved in the first step of *S. aureus *biofilm formation via attachment to the ECM. These include the fibrinogen binding proteins: clumping factor A (ClfA) and ClfB (~95 kDa) [40-43]; and the fibronectin binding proteins: FnBPA and FnBPB (~100 kDa) [35,36,44]. MRCRAMMs allow staphylococci to adhere to a range of cell lines and because FnBPs also bind host serum proteins that coat medical implants they can also mediate adherence to these devices [40,42,45]. 

**Fig. (2):**
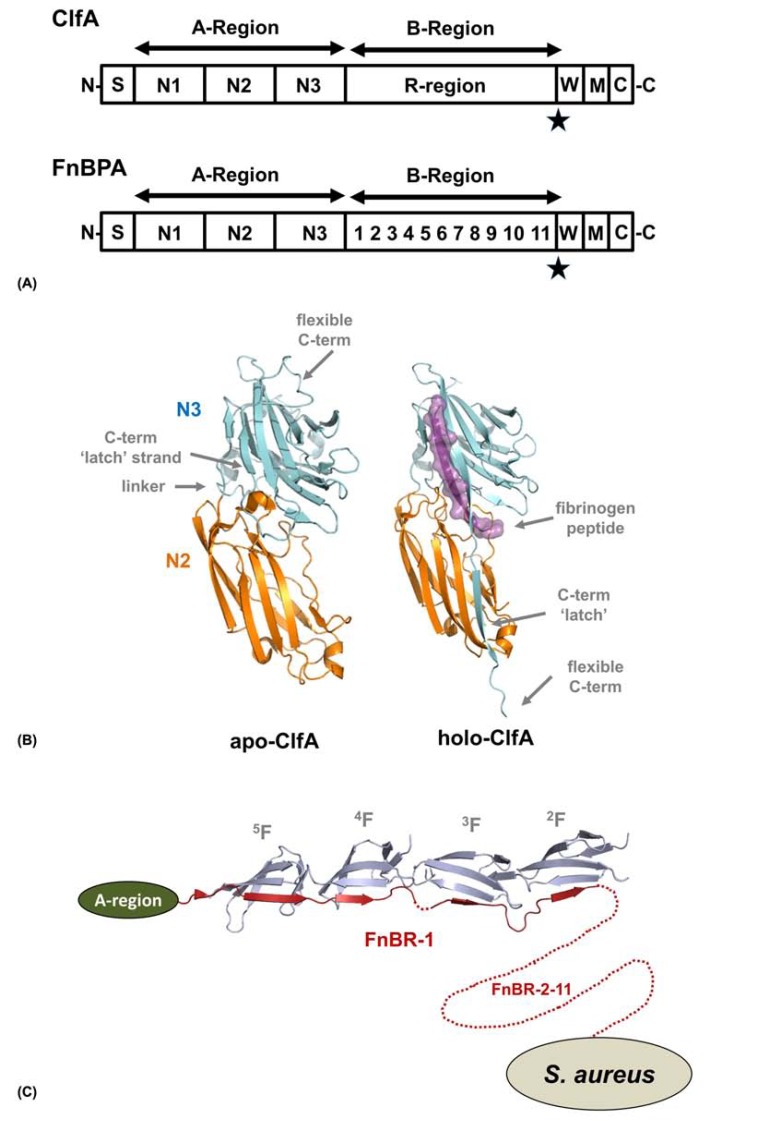
**Adhesive mechanisms of MSCRAMMs.** (A) Schematic of *S. aureus* ClfA and FnBPA adapted from [46]. The N-terminal signal sequence (S) is followed by the A-region, B-region and at the C-terminus is the cell wall anchoring region containing the cell wall sorting region (W) containing the LPXTG motif (star), membrane-spanning hydrophobic domain (M) and the cytoplasmic positively charged C-terminal tail (C). The fibrinogen binding A-region of both ClfA and FnBPA contain three domains (N1-N3). The B-region of ClfA (R-region) is composed of mainly serine and aspartate residues whilst in FnBPA this is made up of 11 fibronectin binding domains (FnBDs: numbered 1-11). (B) Crystal structure of *S. aureus* ClfA with and without a fibrinogen peptide bound. (C) Model of the FnBR-1 region (residues 508-546) of FnBPA in complex with fibronectin (^2^F1^3^F1^4^F1^5^F1). The A-region is shown as a schematic and the FnBR-2-11 region is shown as dashed line (not to scale).

MSCRAMMs are modular in nature and contain an N-terminal signal sequence followed by an extensive repeat/non-repeat region and a C-terminal region for cell wall anchoring [46] (Fig. **2A**). The repeat/non-repeat region can be further divided into an A-region and B-region. The A-region contains ligand binding domains (N1-N3) whilst the role of the B-region is to project the adhesive domains away from the bacterial surface, although they also contain unknown functions. In FnBPs, the B-region in addition harbours the fibronectin binding activity. 

The B-region of ClfA/B is composed of mainly serine and aspartate residues (R-region) and it has been shown that residues 229-545 of ClfA (N2, N3 domains) are sufficient to retain the binding activity for the C-terminal region of the fibrinogen γ-chain (K_d_ 657 nM measured by isothermal calorimetry) [47]. The N1 domain is cleaved by the *S. aureus *metalloprotease autolysin [48] and recombinant ClfA/B expressed in *E. coli *have unstructured N1 domains that are degraded by endogenous proteases prior to purification [49]. The crystal structure of apo-ClfA (pdb: 1N67) was solved in Sthanam Narayana’s group and consists of residues 221-559 [50] with the N2 and N3 domain connected by a short linker (Fig **2B**). Each domain is dominated by β-sheet secondary structure that folds into a variation of the IgG fold. This DEv-IgG fold, named so because two additional strands (D’ and D’’) are inserted between the D and E strands of the standard IgG fold, was first identified in the collagen binding MSCRAMM, Can (pdb: 1AMX) [51], but has since been observed in other systems [52,53]. Cna contains a number of intramolecular isopeptide bonds [54], which are now recognised to be a functionally important feature of many surface proteins in Gram-positive bacteria and have been implicated in stabilizing structures, helping to withstand mechanical stress and facilitating biofilm formation [55]. 

In the crystal structure of ClfA in complex with a 13 residue fibrinogen-derived peptide (pdb: 2VR3), the ligand is recognized and binds along the interface between the N2 and N3 domains [47]. Comparisons between the two crystal structures of ClfA are overall very similar, although in the apo-structure the C-terminal residues fold back into the ligand binding site within the N3 domain [50], whilst in fibrinogen bound ClfA, this sequence crosses over and forms an inter-domain β-sheet in N2. The peptide forms a parallel β-sheet complementation with the G strand of the N3 domain and buries ~1800 Å^2^ of surface area. Ganesh *et al.* [47] have also shown that a mutant of ClfA that contains a disulphide bridge that covalently locks the C-terminal ‘latch’ across the N2 domain binds to fibrinogen with equivalent affinities to a wild-type construct. This suggests that ClfA does not require an ‘open’ conformation to bind its ligand. This is possibly a consequence of ClfA recognizing just the last few residues of the fibrinogen γ-chain that may be threaded into the binding site. 

The A-region of FnBPA/B can also bind the C-terminal region of the fibrinogen γ-chain and likely recognizes this ligand in a similar fashion to ClfA/B [47,56]. The B-region of FnBPs is intrinsically unstructured and contains 11 (FnBPA) or 10 (FnBPB) fibronectin binding repeats (FnBRs) that interact with consecutive fibronectin type I (F1) region in the N-terminal domain of fibronectin [57,58]. Furthermore, in FnBPA, FnBR-1, -4, -5, -10 and -11 bind with dissociation constants in the nM range, whilst the others bind with lower affinities [57]. Jennifer Pott’s group described the NMR structure of a peptide from the *Streptococcus dysgalactiae *FnBP in complex with the fibronectin module pair ^1^F1^2^F1 (pdb: 1O9A) and showed how these class of proteins interact with fibronectin through a tandem β-zipper mechanism [59]. More recently her group has also presented the crystal structure of peptides corresponding to FnBR-1 (residues 508-546) and FnBR-5 (residues 639-672) of FnBPA in complex with ^2^F1^3^F1^4^F1^5^F1 (pdb: 3CAL, 2RL0, 2RKY, 2RKZ) [58]. Each FnBR binds to four consecutive F1 domains, where they form an antiparallel β-strand along strand E of the triple-stranded (strands CDE) β-sheet (Fig. **2C**), again as a tandem β-zipper. Although these interactions are solely mediated by β-sheet interactions, there is ~4300 Å^2^ of surface area buried in each of the FnBR/^2^F1^3^F1^4^F1^5^F1 complexes. It is likely that the interaction with fibronectin is multivalent, binding six to nine fibronectin copies per FnBPA/B molecule [60,61] and taking this into account, the burial of surface area increases to between 26000 to 36000 Å^2^ [58]. 

### 2.2 Serine-Rich Repeat Containing Fimbriae

Fimbriae-associated protein 1 (Fap1) was identified by Paula Fives-Taylor’s group whilst studying novel adhesive proteins from the Gram-positive *Streptococcus parasanguis *FW213 [62]. Sanguis streptococci are primary colonizers of the oral cavity in humans and specific initial attachment is mediated via the salivary components that interact directly with the tooth surface [63]. Furthermore, these streptococci also behave as ligands for additional oral bacterial species and form the substrata within a biofilm on the surface of the teeth: dental plaque [64]. In addition to having a major role in caries and periodontal diseases, these bacteria can also colonize native and prosthetic heart valves and are a common cause of endocarditis [4,65-67].

**Fig. (3):**
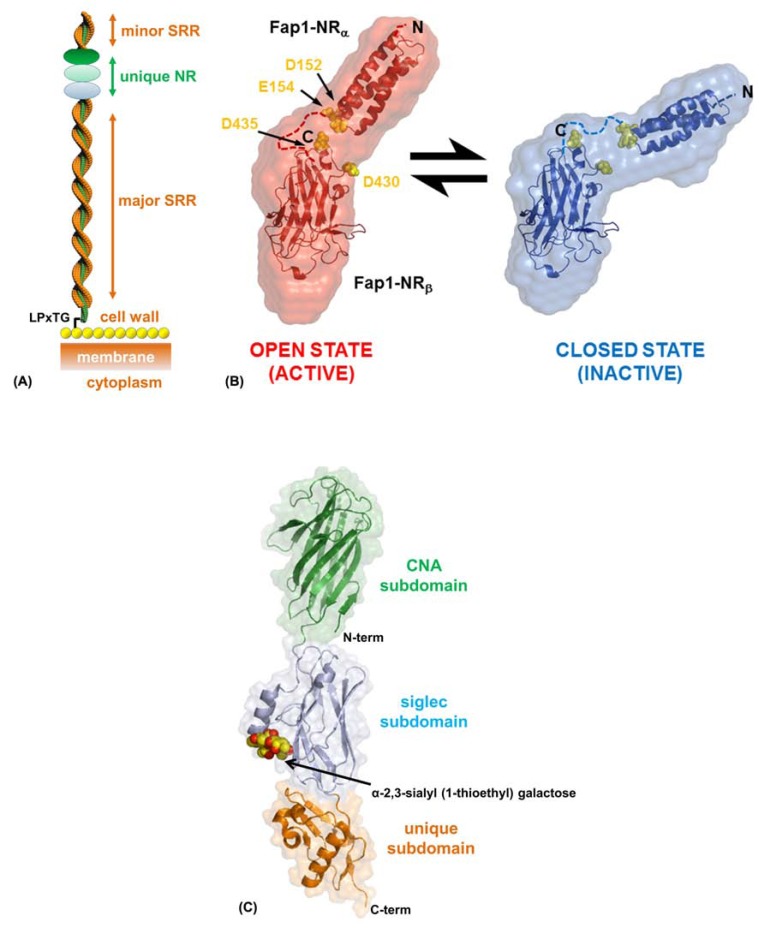
**Adhesive mechanisms of SRRPs.** (A) Schematic representation of a mature SRRP. The N-terminal unique adhesive region is projected away from the cell wall via the extensive SRR region. There is also a minor SRR at the N-terminal pole. The C-terminus is attached to the cell wall peptidoglycan (yellow spheres) through an LPxTG anchor sequence. (B) Conformations of the ‘open’ and ‘closed’ states of *S. parasanguinis* Fap1-NR. SAXS electron densities are shown as envelopes, coloured red (pH 5) or Blue (pH 8), and the structures have been docked into the maps. Acidic residues at the inter-subdomain boundary are highlighted as yellow spheres. (C) Crystal structure of the carbohydrate bound *S. gordonii* GspB_BR_.

Fap1 is a ~200 kDa surface fibre which is essential for fimbrial biogenesis, adhesion and biofilm formation [62,67-70]. Analysis of Fap1 showed it to be composed of an N-terminal signal sequence, followed by a short stretch of (E/V/I)S dipeptide repeats, a unique adhesive region, a much longer dipeptide repeat region and finally at the C-terminus an LPxTG cell wall anchor sequence [68]. The presence of such extensive regions of alternating serine dipeptide repeats (~80% of the overall sequence), which are O-glycosylated through the serine residues [53,67,70-76], has led to Fap1 being termed a ‘serine-rich repeat glycoprotein’ (SRRP). Over the last decade new SRRPs have been discovered and this ever expanding family of Gram-positive bacterial fimbriae now also includes *Staphylococcus aureus* SraP [77], *Staphylococcus agalactiae *Srr-1 and Srr-2 [78-80], *Streptococcus gordonii* GspB [52,81], *S. gordonii* Hsa [82], *Staphylococcus saprophyticus* UafB [83], *Streptococcus pneumoniae* PsrP [84-87], and* Streptococcus sanguinis* SrpA [88]. Primary sequence analysis of this family demonstrates the same overall arrangement within these very large macromolecules, although they each retain unique adhesive features (Fig. **3A**).

SRRPs are glycosylated in the cytoplasm [69,89-91] before being exported to the cell surface via the SecA2/Y2 accessory secretory pathway [72,89,92]. Structures of the unique adhesive region of Fap1 have been recently published (pdb: 2X12, 2KUB, 3RGU) and using structural and biochemical techniques it has been possible to model the overall architecture of the SRR (Fig. **3A**) [53,93,94]. In mature Fap1 the extensive major SRR region forms a super-coiled structure which projects the N-terminal adhesive domain away from the cell surface, with the extensive glycosylation protecting this highly extended region from proteolysis. We have performed biophysical analysis of short synthetic SRR peptides based on Fap1 that show they are unstructured and this suggests the importance of glycosylation in the correct folding of SRRPs (unpublished data).

The ‘non-repeat’ region of Fap1 (Fap1-NR) was characterized as an adhesive domain after it was shown to interact with an *in vitro* tooth model: saliva-coated hydroxylapatite (SHA) [95]. Furthermore, these adhesive properties can be modulated by pH with a much greater affinity observed under acidic conditions [53]. Fap1-NR can be further subdivided into an α-helical (Fap1-NR_α_) followed by a β-sheet (Fap1-NR_β_) region. NMR analysis and SHA assays show that whilst both are involved in binding to a yet unknown host ligand in the salivary pellicle, it is the helical subdomain that directs the pH-mediated effects [53]. SAXS data from Fap1-NR at pH 5.0 and pH 8.0 reveals a ‘boomerang’ shape that ‘opens’ under acidic conditions (Fig. **3B**). Comparison of the crystal structure of Fap1-NR_α_ at pH 5.0 [93,94] and the NMR structure at pH 8.0 [53] shows no significant differences, although docking of these structures and the crystal structure of Fap1-NR_β_ into these low resolution envelopes identifies a mechanism of adhesion modulated by electrostatics (Fig. **3B**) [53,93]. 

In Fap1-NR Asp152, Glu154, Asp430 and Asp435 pack against one another within the inter-subdomain face and this has the effect of raising the local pKa of these carboxylate groups so that they are protonated at a higher pH (Fig. **3B**) [93]. These subdomains are connected by a 27 amino acid linker and NMR relaxation data indicates that there is independent motion in both [53]. Therefore the Fap1-NR ‘open/active’ conformation results from a rearrangement of this region and the potential for intra-Fap1-NR salt bridges to form. The normal resting saliva pH in humans is between 6.5 and 7.1, however, after the ingestion of fermentable carbohydrates, microbial acid production can lead to a drop in plaque pH to below 5.0. Unlike some streptococci, *S. parasanguis* does not have an acid tolerance response [96,97] and instead it shuts down its metabolic functions [6]. So Fap1 presents a mechanism of reversible adhesion for *S. parasanguis* under metabolically active neutral/alkali conditions, but at low pH whilst metabolically dormant, Fap1 provides a much higher affinity for the salivary pellicle and in turn the mature biofilm which protects *S. parasanguis* [53]. 

Work in Carlos Orihuela’s group has shown that the SRRPs PsrP from *Streptococcus pneumoniae*, SraP* from Staphylococcus aureus* and GspB from* Streptococcus gordonii* strain M99 can all mediate inter-bacterial biofilm interactions in the lungs of infected animals via their unique non-repeat regions, in addition to the well documented host adhesive properties [98]. Recently the crystal structure of the GspB adhesive region (GspB_BR_) from *S. gordonii* was solved (pdb: 3QC6, 3QC5, 3QD1) in Tina Iverson’s group, and this has exemplified how the basic SRRP architecture can accommodate a plethora of different functions [52]. GspB is an important adhesive structure of *S. gordonii* M99 and loss of expression leads to a noticeable effect on the pathogenicity of this strain [99]. GspB binds to human platelets through the membrane glycoprotein GPIbα [100] and specifically it recognizes sialyl-T antigen, which is one of the major carbohydrates of GPIbα [101]. 

The crystal structure of apo-GspB_BR_ is composed of three adjacent domains and has been described as being like beads on a string [52]. The N-terminal subdomain is reminiscent of an Ig-like fold and has high tertiary homology with the A-region of the *S. aureus *Cna [51] and *S. parasanguinis* Fap1-NR_β_ [53]. Whilst the C-terminal subdomain has a unique fold, the central subdomain has structural homology with eukaryotic Siglecs, which are involved in the binding of carbohydrates. The crystal structure of GspB_BR_ in complex with an analogue of sialyl-T antigen confirmed this function (Fig. **3C**). A deviation of the subdomain orientation within these two GspB_BR_ structures suggests that these regions display independent motion. Furthermore, the modular nature of these motifs hints at multiple functions, which may be independent or cooperative as within Fap1-NR [97]. Mutations within the lectin domain of GspB_BR_ cause a significant reduction in bacterial densities within kidneys and spleens and so it is likely that it is this region that facilitates the main *S. gordonii* M99 interactions in endocarditis [96]. However, these additional domains may be involved in other virulence strategies such as mediating GspB-GspB interactions during later stages of biofilm development.

## 3 PROTEIN MEDIATED INTER-BACTERIAL INTERACTIONS

Once prokaryotes have formed a tight adherence to a surface they begin to divide and scan their local environment for interactions with siblings and genetically distinct partners. This marks the initiation of the three-dimensional architecture of a biofilm. At this stage cellular communications are mediated by surface exposed protein interactions and while our biochemical and genetic understanding of these processes are quite detailed, high resolution structural information is lacking. Again in this section, structures will only be described which allow an atomic understanding of early biofilm formation. A recent crystal structure of the B-region of *S. aureus *SasG (PDB: 3TIP, 3TIQ) from Jennifer Potts group gives a tantalizing suggestion of how these classes of proteins may form protein-protein or protein-saccharide/ DNA interactions through the sequestration of Zn^2+^ [102], yet as no high-resolution data is available this will not be described. At the time of writing this review, only three bacterial structures had been deposited in the PDB that describe the atomic details of protein-mediated interactions which allow biofilms to begin and take shape. These will be discussed here. 

### 3.1 The *Haemophilus influenza* Hap

Autotransporters (ATs) belong to the type V secretion system family and account for the largest number of exported proteins from Gram-negative bacteria [103]. ATs contain a single polypeptide chain consisting of a C-terminal outer membrane β-barrel pore through which the N-terminal passenger domain is transported. The N-termini contain a perisplasmic signal sequence that directs them through the Sec apparatus, and whilst for a long time it was believed that insertion of ATs into the outer membrane utilized the Omp85 superfamily [104], new data suggests that they use a dedicated translocation and assembly module (TAM) [105]. Passenger domains, which usually fold into a β-helix like structure, are important virulence factors in Gram-negative pathogens and upon export (and often cleavage from the surface) they can function as proteolytic enzymes, adhesins, invasins and toxins [106]. A subset of the AT family are called the self-associating autotransporters (SAATs) and they share sequence homology and mediate inter-bacterial aggregation [107]. These SAATs include AIDA, Ag43, and TibA from *Escherichia coli* and recently Joseph St Geme’s group has published the structure of Hap from *Haemophilus influenza* (pdb: 3SYJ) and presented a general model for SAAT-mediated bacterial auto-aggregation [108,109].

**Fig. (4):**
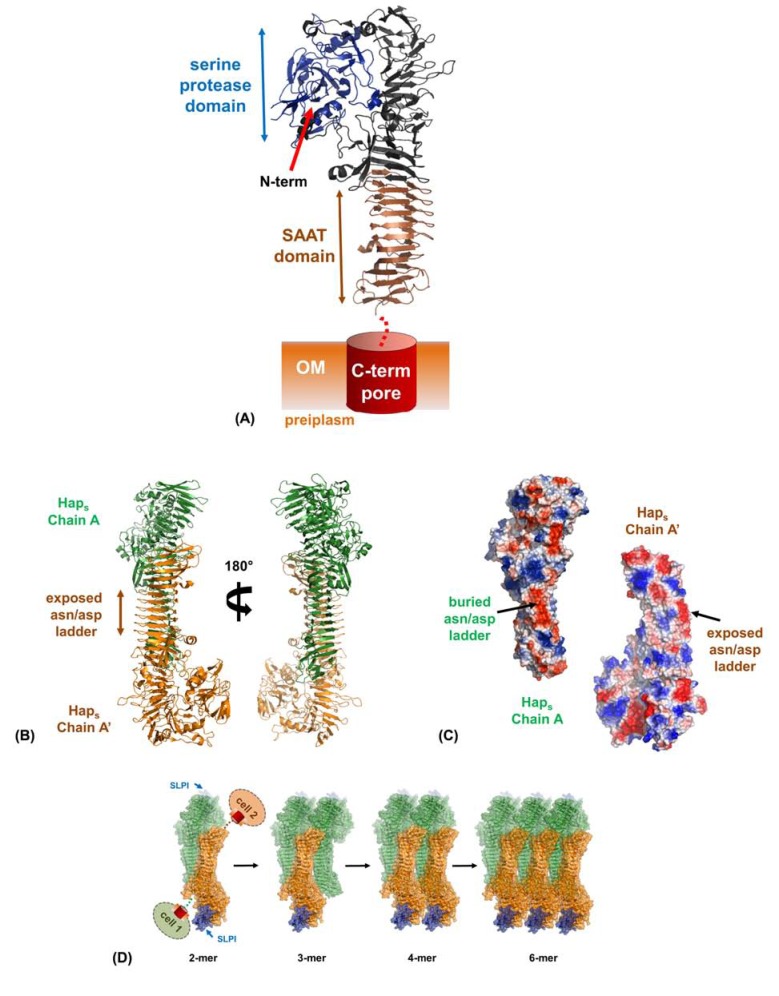
**Hap-Hap mediated biofilm formation.** (A) Crystal structure of the *H. influenza* Hap passenger domain (Haps) with the C-terminal pore shown as a schematic. (B) Crystallographic relationship between Hap_s_ molecules in the crystal. The interface of a dimer of Hap_s_ in *trans* (coloured orange and green) shows that a run of Asp/Asn residues (the Asp/Asn ladder) from one subunit packs against a complimentary but alternative surface of the other molecule (shown as electrostatic surfaces : dark regions). (C) The remaining Asp/Asn ladder from the latter molecule of the dimer is still accessible and with the burying of hydrophobicity, translations of these dimers can lead to great multimers forming. The modelled SLPI bound to each serine protease domain is coloured blue.

*H. influenza* is an agent of bacteremia, pneumonia and acute bacterial meningitis. Hap is involved in the adhesion of *H. influenza* to epithelial cells and extracellular matrix proteins, invasion of epithelial cells and inter-bacterial aggregation during early biofilm development [110]. The N-terminus contains the usual signal peptide, followed by a passenger domain (Hap_s_) consisting of a serine protease, an ECM-binding/SAAT domain and finally the C-terminus comprises of the outer membrane β-barrel motif (Fig. **4A**). The ECM-binding domain is responsible for recognizing fibronectin, laminin and collagen IV [111] and the SAAT region is involved in interactions with epithelial cells [112] and also Hap-Hap mediated biofilm processes [112,113]. The crystal structure of the full Hap passenger domain is described as a ‘Dane Axe’-like assembly (Fig. **4A**). The main spine of the structure is formed from a β-helix and has at the C-terminus the SAAT domain, overlapped with the ECM-binding domain. The N-terminal region of the β-helix acts as a scaffold for the positioning of the serine protease at the extreme N-terminus of the passenger domain.

The SAAT domain of Hap is entirely β-helical and displays a hydrophilic edge of stacked Asn/Asp residues [109]. Furthermore, this region is unusually straight and forms a striking triangular prism composed of approximately nine strands per face. Remarkably, the crystal lattice packing relates two molecules in *trans* configuration by a crystallographic 2-fold screw axis (Fig. **4B**). Inter-dimer interactions are mediated in the main by a ladder of Asn/Asp hydrogen bonds from one monomer to the face of another, with also some burial of hydrophobicity. A secondary dimer interface is formed by a region flanking either end of the SAAT domain. As this dimer forms with a single monomer’s Asn/Asp ladder, the other remains exposed for higher ordered oligomerization. Further symmetry relations build an array of multimerized Hap molecules with the N-terminal β-helix facilitating significant inter-molecular interactions (Fig. **4C**). These inter-Hap mediated interfaces have been rigorously tested using a number of mutants in a self-association assay and this presents a valid model for Hap-Hap biofilm activity. Once this initial dimer has formed *in vivo *it likely acts as a nucleant for the assembly of mega-Dalton complexes of immense stability that overcomes the repulsive force between bacteria [109]. 

In the work by Meng and colleagues [109], they were able to go one step further and model the functional modulation of the Hap biofilm. The serine protease is autoproteolytic and can release adjacent Hap passenger domains from the bacterial surface to regulate host and inter-bacterial adhesion [112,113]. Interestingly this proteolytic activity is inhibited by the secretory leukocyte protease inhibitor (SLPI) [114], which is present in the upper and lower respiratory tract [113]. In the model of Hap-Hap multimerization, the protease domain is still partially solvent exposed and superimposition with the elastate/SLPI structure [114] permitted the positioning of SLPI with respect to Hap (Fig. **4C**) [87]. This suggests that in the absence of SLPI the serine protease activity will release adjacent Hap from the *H. influenza* surface, whilst in its presence this activity is revoked resulting in oligomerization and inter-bacterial aggregation. 

### 3.2 The *Escherichia Coli* Common Pilus

In 2001 Timo Korhonen’s group published data on a novel fimbria isolated at low temperatures from* Escherichia coli* associated with newborn meningitis and septicaemia (NMEC) [115]. This was called the meningitis associated and temperature regulated (Mat) fimbria, although over the past decade Jorge Girón and José Puente’s groups have shown it to be ubiquitous across most *E. coli* strains and it is now usually referred to as the *E. coli* common pilus (ECP) [116-119]. *E. coli *are primarily commensal colonizers of the human and other animal bowels and they contribute to a healthy immune system of the host. There are also a number of virulent strains that can cause diarrheal diseases such as hemorrhagic colitis [120]. Furthermore, if they enter extraintestinal sites these strains can also lead to neonatal meningitis, urinary tract infections, sepsis, and pneumonia [121].

**Fig. (5):**
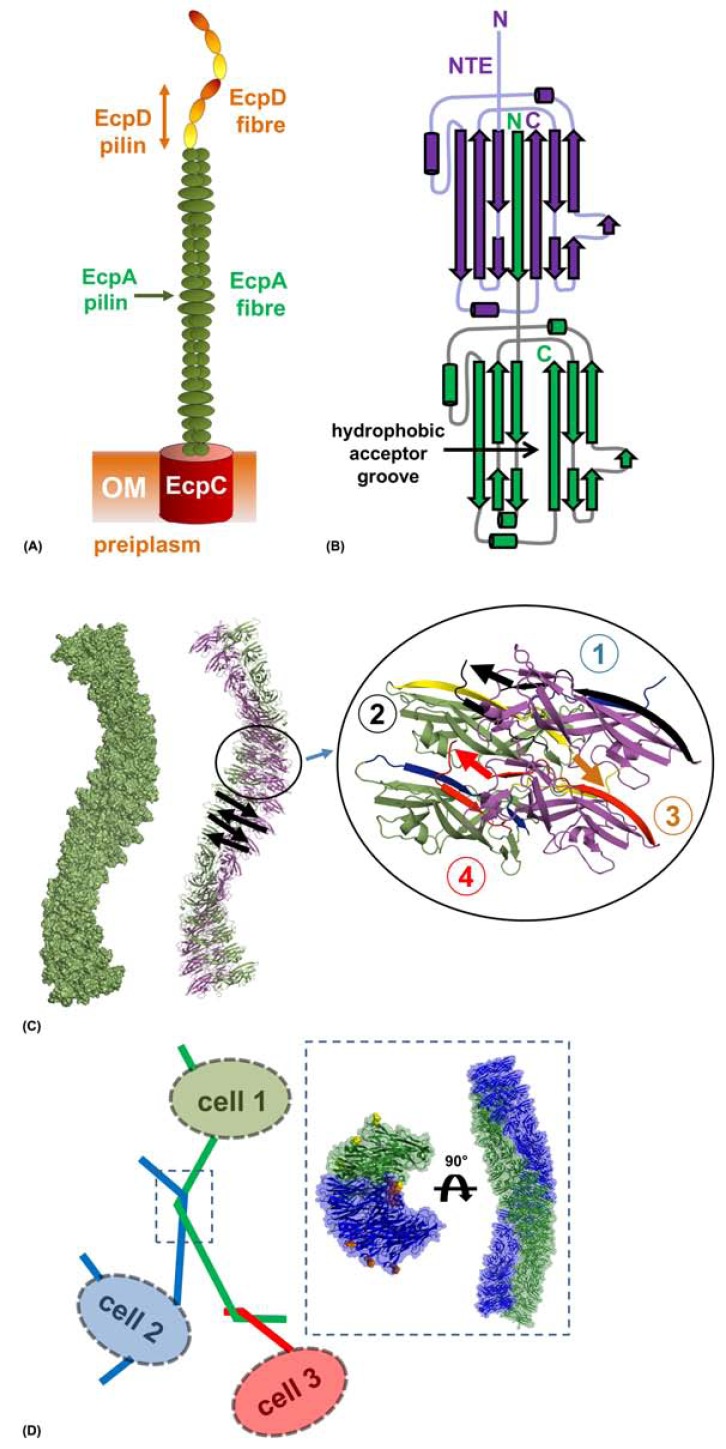
**ECP-ECP mediated biofilm formation.** (A) Model of the *E. coli* common pilus displayed on the cell surface with the usher pore (EcpC), the major pilus (EcpA) and the polymerized tip adhesin (EcpD) annotated. (B) Schematic representation of donor strand exchange between EcpA domains. One EcpA subunit (green) donates its N-terminal extension (NTE) to the adjacent EcpA subunit (purple) where it lines the hydrophobic groove. (C) Atomic model of ECP fibres. A single fibre from crystals of EcpA is shown as a surface (left) and as a cartoon with adjacent subunits coloured green and purple. The direction of polymerization in the fibre is shown with black arrows. Four subunits have been expanded to highlight the zig-zagging and helicity of EcpA along the fibre length. (D) Representation of ECP-ECP mediated antiparallel interactions. Cells 1-3 are shown with two ECP fibres intertwined by a half helical turn. One of these regions has been boxed and expanded, showing the atomic model which describes this event. Two antiparallel entwined ECP are shown from the side and top.

ECP fibres are assembled via a variant of the CU pathway and as with all members of this superfamily, filaments are formed from polymerisation of several different/identical pilin domains (Fig. **5A**) [18,122]. The tip of ECP is uniquely composed from a polymerized array of a novel 60 kDa adhesive domain EcpD, which recognizes an unknown ligand on the host cell surface [122]. The majority of ECP is composed of an 18 kDa domain called EcpA [115,116], which functions in binding hydrophobic surfaces and mediating inter-bacterial aggregation in early biofilm formation [122,123].

The crystal structure of EcpA (pdb: 3QS2, 3QS3) from uropathogenic *E. coli* (UPEC) has been recently solved by our group [122]. Like other CU major pilin domains, EcpA is formed from an incomplete Ig-like fold, where an adjacent molecule in the fibre donates its N-terminal strand (N-terminal extension; NTE) to fill a hydrophobic groove running along the full length of EcpA, completing the very stable Ig-like motif (Fig. **5B**). EcpA is fashioned from approximately 50% hydrophobic residues and the surface is scattered with hydrophobic patches including a number of aromatic residues. This likely promotes a less-specific contact with a wide range of hydrophobic substrates and polymers. 

Pili assembled via the CU pathway vary greatly in size and function. Afa-III fimbrils are very flexible and are composed of a head-to-tail polymerisation of subunits ~2 nm in diameter [124], whilst type I pili are rigid structures formed by the major pilin molecules packing about a central axis giving rise to a hollow fibre of ~7 nm in diameter [125]. ECP are quite flexible with a width ~6 nm, which consistently varies along the fibre length [122]. Crystal structures of donor strand complemented EcpA reveals a fibre-like arrangement of domains with single filament dimensions matching those observed under EM (Fig. **5C**) [122].

EM images of *E. coli* producing ECP show these fibres form a mesh that encapsulates the whole microcolony. ECP interacts with itself through pili crossing over one another, parallel fibre entwining and antiparallel entwining [116,122]. The crystal lattice of EcpA also revealed an intertwining of antiparallel fibres giving rise to a super helical diameter of ~12 nm (Fig. **5D**). Inter-ECP interactions are conducted in the main via burial of the loop residues Ala147, Val148 and Thr149 across the central axis and mutations of these residues to bulky amino acids results in dramatic reductions in cell mass in biofilm assays. EcpA is highly conserved amongst a range of other enteric bacterial species including *Serratia proteamaculans*, *Serratia odorifera*, *Klebsiella sp.*, *Klebsiella pneumoniae*, and *Enterobacter cancerogenus*, which suggests a role for ECP in establishing contacts between multiple species [122]. 

### 3.3 *Streptococcus parasanguis* Fap1

Fap1 is a model system to study the biogenesis, export and general architecture of SRRPs; however, in its own right it displays some very unique properties. As has been detailed above, the binding of Fap1 to SHA is affected greatly by the pH of the environment and this can be attributed to a survival mechanism of *S. parasanguis* when experiencing long periods of acidity. Moreover, there is much evidence that Fap1 also functions in mid and later stages of biofilm development. The heavy glycosylation has been implicated in some of these processes [67] and the extreme N-terminal SRR region may also contributes here. This role likely plays out via hydrophobic stacking of saccharides and/or recognition by lectins within the mature biofilm matrix. Interestingly though, visualization of Fap1 displayed on the surface of *S. parasanguis* using EM also shows a pH-dependence to the auto-aggregation of these fibres, specifically at their N-terminal pole [53]. In addition, biofilm assays performed with *S. parasanguis* over a range of pH values show a clear increase in cellular mass correlated to acidity, mirroring the binding of Fap1-NR to SHA [53,93].

**Fig. (6):**
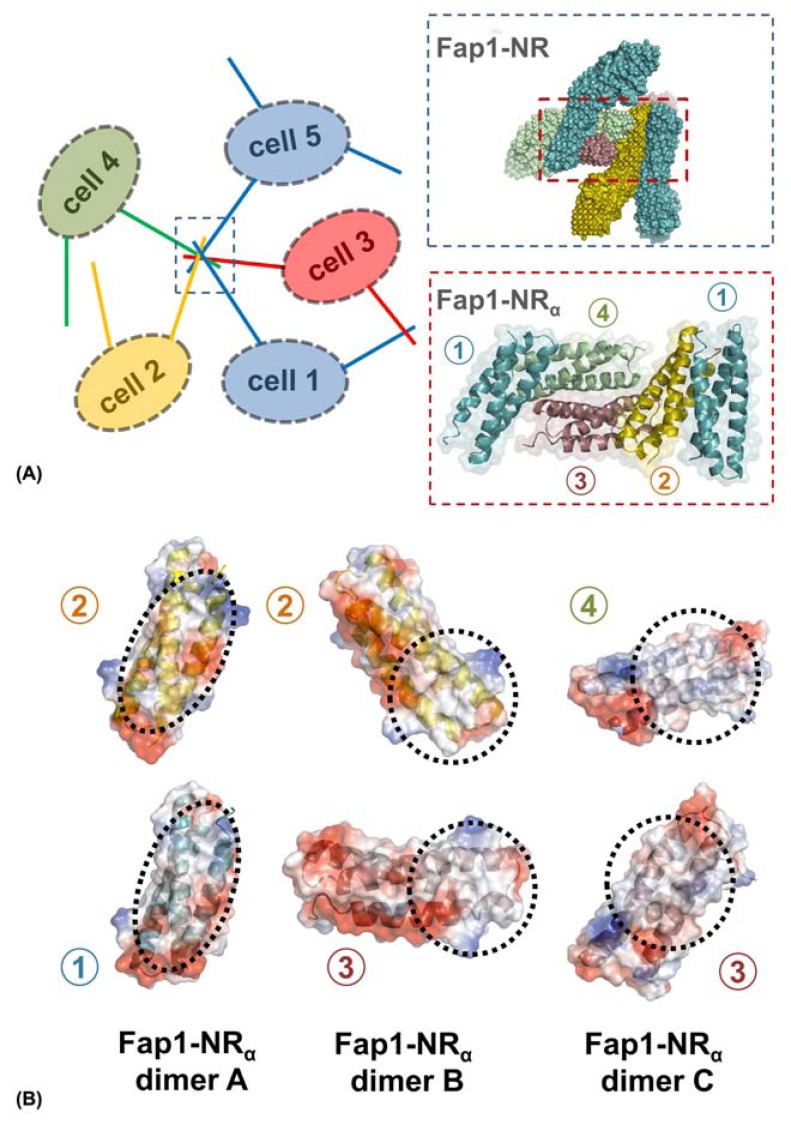
**Fap1-Fap1 mediated biofilm formation.** (A) Representation of Fap1-NR_α_ mediated Fap1-Fap1 interactions. Cells 1-5 are displayed with Fap1 filaments on their surfaces. A number of these fibres are interacting via their tips, and this has been expanded to show the surfaces of Fap1-NR orientated based on the Fap1-NR_α_ crystal structure. The Fap1-NR_α_ region has been further expanded to show the packing of five molecules from these crystals. The numbers represent the unique molecules of the asymmetric unit. (B) Fap1-NR_α_ crystals are formed from 3 types of dimer (A-C). Each dimer interface is drawn as an electrostatic surface and the specific contact areas are circled.

The crystal structure of Fap1-NR_α_ has been very insightful in terms of our understanding of how Fap1 may form inter-filamentous aggregation via the N-terminal tip (Fig. **6A**) [93]. The arrangement of Fap1-NR_α_ in the crystal lattice clearly demonstrates an order that can accommodate full Fap1-NR domains and allow the major SRR region to project back to a bacterial surface from multiple orientations [93], in a similar fashion to that observed under EM [53]. No inter-domain salt bridges form within these aggregates, but some of these dimers are formed solely by the burial of hydrophobic surfaces, whereas others also involve patches of negative charge (Fig. **6B**). This is consistent with the observations that Fap1 tip interactions can form at pH 8.0 but become much more prevalent at pH 5.0 [53]. Furthermore, within this aggregate a number of the Fap1-NR host-binding sites are not fully occluded and may allow the dual role of salivary pellicle recognition and inter-cellular negotiation. 

## 4 EPS-MEDIATED INTERACTIONS

In the middle stages of biofilm formation, cells develop into a three-dimensional array and start producing the sticky conglomerate EPS, of which the composition can be very diverse between different species. On average 90% of a biofilms dry mass will be accounted for by the EPS, and this is the immediate environment which these prokaryotes sample [14]. Biofilms can take on a plethora of architectures and this is also crucially dependent on the localized production and quantity of EPS. The biofilm matrix is mainly composed of exopolysaccharides, DNA, protein and lipids. Although no experimental high resolution structures are available for these components, due to the nature of polysaccharides, DNA and some of the protein based EPS (i.e. amyloids) it is possible to use modelling techniques (i.e. X-ray fibre diffraction [126,127] and NMR [128,129]). However, whilst this information is invaluable, from an atomic perspective we know very little about how the matrix interacts within itself. 

The major contributors of the bacterial EPS matrix are polysaccharides [130] and many mutants that cannot synthesize exopolysaccharides are unable to form mature biofilms [131,132]. These are mainly heteropolysaccharides, attached to the cell surface forming long linear and branched structures, which establish a complex mesh. A number of well-known exopolysaccharides include alginate, Pel and Psl from *Pseudomonas aeruginosa *[133]. Depending on the composition of saccharides these polymers can display more or less hydrophobicity or ionic nature. This gives rise to a very large and durable structure with variable functionality. These polysaccharides can interact with biotic/abiotic surfaces, further increasing the affinity of the bio-mass whilst also cross linking the sessile cells through sugar-sugar and sugar-lectin contacts. Very recently the first direct evidence for interactions between the T4P PilA and exopolysaccharides of *Myxococcus xanthus* were observed under EM [134]. Some biofilms also have a very hydrophobic nature due to the lipidation of carbohydrates and this property is essential for the adherence of *Thiobacillus ferrooxidans* to pyrite surfaces [135]

Nucleic acids in the form of DNA can be in abundance in biofilms, although whilst in *S. aureus* it is a major component of the matrix structure, it is less so in *S. epidermis* [136]. The source of extracellular DNA (eDNA) comes from the lysis of bacteria within the biofilm and it has been shown that *Enterococcus faecalis* are able to specifically lyse a fraction of the bacterial population using a protein GelE [132]. In *P. aeruginosa* eDNA is used to connect cells and treatment with DNase inhibits biofilm formation [137,138], whilst in *Bacillus cereus* eDNA has also been shown to function as an adhesin [139]. Furthermore, DNA is a versatile molecule for specific recognition by proteins such as the T4P of *P. aeruginosa *[140], resulting in a heterogeneous protein-DNA network. This localization of eDNA also represents a potential reservoir for the horizontal transfer of genes and the increased virulence/persistence of strains within bacterial communities. 

Another important type of macromolecule involved in the maturation and shaping of biofilms are microbial amyloid fibres [141]. Amyloids were historically thought to be a consequence of protein misfolding in neurodegenerative diseases such as Alzheimer’s and Parkinson’s [142], but it is now understood that they fulfil an important role in a number of organisms. Amyloid are β-strands that are orientated perpendicular to the fibre axis [143] and the Enterobacteriaceae curli are a model system to study this family [144,145]. Small curli subunits (CsgA) are secreted to the extracellular space where they polymerize into the amyloid and contribute the major proteinaceous component of the *E. coli *and *Salmonella enterica* serovars Typhimurium biofilm matrix. Curli are crucial in these biofilms and mediate initial surface attachment and provide a scaffold for the community [144,146,147]. Whilst highly stable models of bacterial amyloids have been proposed [143], the molecular details that underlie these processes are poorly understood. 

## 5 STRUCTURING AND DISPERSAL OF BIOFILMS

The controlled restructuring of biofilm architecture results in a heterogeneous arrangement of matter and the production of cavities and channels. This process can be undertaken by both targeted cell lysis and cell dispersion. Another process that is closely linked to these restructuring events is the partial breakdown of the matrix to allow the release of cells, which are free to migrate and inhabit new environments, i.e. when nutrients become limiting and when waste products accumulate [148]. These effectors or dispersins can often have enzymatic activity towards polysaccharides [149], proteins [150] or DNA [151]; or function as surfactant-like molecules [152,153]. Furthermore, bacteriophages also play an important role and can induce cell death and provide enzymes that help dissolve the biofilm matrix [154]. 

*Aggregatibacter actinomycetemcomitans* is a Gram-negative non-motile commensal of the oral cavity, associated with gum disease. Dispersin B is an extracellular enzyme (PDB: 1YHT) [155] secreted by *A. actinomycetemcomitans* and can degrade matrix polysaccharides [156]. This is a classic example of enzymatic disruption of the biofilm matrix. Whilst dispersin B has been identified as an essential factor for the dispersal of *A. actinomycetemcomitans* biofilms [157], it has also been demonstrated to induce dispersal of a range of other bacteria that contain an poly-*N*-acetylglucosamine substrate [158,159]. 

An alternative strategy of restructuring/dispersal is exemplified by the *P. aeruginosa* rhamnolipid [153,160] and staphylococcal phenol-soluble modulins (PSMs) [152,161]. The immense stability of bacterial biofilms can be attributed to the extensive burial of hydrophobic material within the matrix. Therefore, surfactant-like reagents such as rhamnolipid and PSMs have the ability to disrupt these interactions in a non-specific manner. PSMs are α-helical, amphipathic peptides ranging from ~20-50 amino acids [162-164] and their expression is controlled through quorum sensing [86]. These are intriguing and novel molecules that have been identified as key contributors to many aspects of the *S. aureus *biofilm maturation process [152] including the formation of channels, biofilm detachment, biofilm expansion and dissemination. 

## 6 THERAPEUTIC TARGETS OF BIOFILM DISSEMINATION

Biofilms represent the dominant microbial life style in aquatic environments, providing nutrients, purification of water, sequestration of carbon and biogeochemical fluxes; i.e. roles that are essential for these and other habitats to exist [165]. Compounds such as Triclosan have been reported to disseminate biofilms in a global fashion by targeting EPS secretion [166]. These reagents are very effective in one sense, but obviously they are highly detrimental to ecosystems and better strategies are needed that target essential processes within specific species of biofilm. 

One point of entry to combat biofilms is to stop them before they can take hold through inhibition of the initial attachment. A number of strategies have been investigated that target both the adhesive mechanisms and also the biogenesis pathways from which these adhesins are produced. An example of the latter are Gram-negative bacteria that use CU assembled pili as adhesive filaments: compounds termed ‘pilicides’ have been shown to disrupt the biogenesis machinery of the type I pilus and inhibit biofilm formation in UPEC [167,168]. Antimicrobial peptides coated on surfaces have also exhibited activity against *S. aureus *and *P. aeruginosa* [169]. It is an essential requirement of modern medical devices to possess antimicrobial properties and it is now evident that both the component material (i.e. copper, gold, zinc and single walled nanotubes) [170,171] and the sub-micropatterning of the surface [172] both dictate bacterial growth. 

Once a biofilm has become established, modern antibiotics have little effect on their displacement [173] and can make them more resilient [174]. The EPS acts as a barrier to drug delivery [175] and quorum sensing within these dense communities can up-regulate gene expression linked to antimicrobial resistance [176]. Therefore two possibilities for therapeutic advances on mature biofilms are available; direct dispersal mechanisms and communication pathways. 

Enzymes perform functions in biofilm restructuring and whilst they are highly specific, they are expensive to produce and can be unstable. Furthermore, in a medical setting they may also cause an unwanted immune response, therefore the direct use of enzymes in therapeutics does not seem viable. Phages, however, have been reported to improve the normal antimicrobial activity in biofilm related infections [177] and moreover, the *Bacillus licheniformis* lipopeptide biosurfactant has been shown to have a marked effect on increasing antibiotic activity against *E. coli* biofilms [178]. Although these strategies are feasible, much development is needed before they can be used in any real applications. Richard Losick’s group have shown that *Bacillus subtillis* release a factor that inhibits the formation of, and initiates the breakdown of existing biofilms [179]. This was identified as a mixture of the amino acids D-tyrosine, D-leucine, D-tryptophan and D-methionine. Furthermore, a number of D-amino acids also have similar effects on *S. aureus* and *P. aeruginosa* biofilms. These amino acids function through incorporation into the cell wall peptidoglycan where they stimulate release of the amyloid TasA from the matrix [180,181]. In addition, another secreted *Bacillus subtillis* compound, norspermidine, seems to act in a complimentary manner by targeting exopolysaccharides [182]. 

Bacteria have developed highly robust cell-cell signalling or quorum-sensing mechanisms. In *S. aureus* quorum-sensing inhibits biofilm development [183,184] via the *agr *(accessory gene regulator) locus, which produces and senses AIP (autoinducing peptide). AIP is an eight residue peptide where the C-terminal five residues form a cyclic thiolactone ring [185]. AIP has long been known to control virulence factor expression but it also mediates biofilm detachment through activation of an EPS proteases [150]. Furthermore, *P. aeruginosa* produce an organic compound, *cis*-2-decenoic acid, which can disseminate established biofilms and also inhibit biofilm development [186]. This may or may not operate through manipulating quorum-sensing pathways, but none-the-less in addition to *P. aeruginosa* this highly potent fatty acid can also disrupt many other Gram-negative biofilms. A number of these natural dispersal mechanisms have been coordinated with current antibiotic therapies and have shown great promise in dissemination of medically relevant biofilms [187]. 

## CONCLUDING REMARKS

Biofilms represent the most frequent mode of growth for many microbes. While headway is being made in understanding their formation and development, we are still far from being able to describe all of these processes from a molecular perspective. As further insights into this complicated life style are made available, both at the atomic and cellular level, new targets to be exploited will arise, giving us a much wider scope to address problematic biofilms.
